# The effect of compliance with PAP therapy on biochemical parameters in patients with obstructive sleep apnea syndrome: A 6-month follow-up study

**DOI:** 10.5578/tt.202401869

**Published:** 2024-03-26

**Authors:** Alperen AKSAKAL, Buğra KERGET, Hatice Beyza ÖZKAN, Ömer ARAZ, Elif YILMAZEL UÇAR, Leyla SAĞLAM, Esra LALOĞLU

**Affiliations:** 1 Department of Pulmonary Diseases, Atatürk University Faculty of Medicine, Erzurum, Türkiye; 2 Department of Biochemistry, Atatürk University Faculty of Medicine, Erzurum, Türkiye

## Abstract

**ABSTRACT**

**
The effect of compliance with PAP therapy on biochemical parameters in patients with obstructive sleep apnea syndrome: A 6-month follow-up study
**

**Introduction:** *The gold standard treatment for obstructive sleep apnea syn- drome (OSAS) is positive airway pressure therapy (PAP) treatments. PAP treatments reduce complications by reducing apnea and hypopnea attacks by creating airflow at a determined pressure. In our study, we aimed to examine the effect of treatment compliance on kidney and liver functions, apnea- hypopnea (AHI) index, and lipid profile of patients diagnosed with OSAS and started PAP treatment.
*

**Materials and Methods:** *Patients who were admitted to the sleep laboratory of our hospital between September 2022 and September 2023 and started PAP treatment after PSG were included in our study. Patients who were called for follow-up six months after the initiation of PAP treatment were divided into two groups according to their compliance with PAP treatment. Patients who used the device for at least four hours per night and more than 70% at night were grouped as PAP-compliant patients, while the other patients were grouped as non-PAP-compliant patients.
*

**Results:** *It was observed that uric acid, BUN, triglyceride, total cholesterol, ALT, GGT, ALP, and AHI levels of the patients who started PAP treatment decreased after six months (p= 0.001, 0.006, <0.001, 0.006, 0.01, <0.001,
*

*
<0.001, <0.001 with). It was observed that HDL cholesterol levels increased (p≤ 0.001). It was observed that the change in uric acid, AHI, total choles- terol, and GGT levels in group 1 (n= 36) patients who were compliant with PAP treatment was statistically higher than in group 2 (n= 30) patients (p≤ 0.001, <0.03, <0.001, 0.008, respectively).
*

**Conclusion:** *Uric acid, total cholesterol and GGT are biomarkers that may increase in OSAS due to intermittent hypoxia with the involvement of other systems. Since a decrease in these biomarkers can be observed in the early period depending on treatment compliance, these biomarkers can be used practically in the follow-up of treatment compliance and treatment efficacy.
*

**Key words:** *OSAS; positive airway pressure therapy; biomarker
*

**ÖZ**

**
Obstrüktif uyku apne sendromlu hastalarda PAP tedavisine uyumun biyokimyasal parametreler üzerine etkisi: 6 aylık takip çalışması
**

**Giriş:** *Obstrüktif uyku apne sendromu (OSAS) için altın standart tedavi pozitif hava yolu basıncı (PAP) tedavileridir. PAP tedavileri, belirlenen basınçta hava akımı oluşturarak apne ve hipopne ataklarını azaltarak komplikasyonları azaltır. Çalışmamızda OSAS tanısı alan ve PAP tedavisi başlanan hastalarda tedavi uyumunun böbrek ve karaciğer fonksiyonları, apne-hipopne indeksi (AHİ) ve lipid profili üzerine etkisini incelemeyi amaçladık.
*

**Materyal ve Metod:** *Eylül 2022 ile Eylül 2023 tarihleri arasında hastanemiz uyku laboratuvarına başvuran ve PSG sonrası PAP teda- visi başlanan hastalar çalışmamıza dahil edildi. PAP tedavisi başlandıktan altı ay sonra kontrole çağrılan hastalar PAP tedavisine uyumlarına göre iki gruba ayrıldı. Cihazı gecede en az dört saat ve %70’in üzerinde kullanan hastalar PAP uyumlu hastalar olarak gruplandırılırken, diğer hastalar PAP uyumlu olmayan hastalar olarak gruplandırıldı.
*

**Bulgular:** *PAP tedavisine başlayan hastaların ürik asit, BUN, trigliserit, total kolesterol, ALT, GGT, ALP ve AHI düzeylerinin altı ay sonra düştüğü gözlenmiştir (p= 0,001, 0,006, <0,001, 0,006, 0,01, <0,001, <0,001, <0,001 ile). HDL kolesterol düzeylerinin arttığı gözlen- miştir (p≤ 0,001). PAP tedavisine uyumlu grup 1 (n= 36) hastalarda ürik asit, AHİ, total kolesterol ve GGT düzeylerindeki değişimin grup 2 (n= 30) hastalara göre istatistiksel olarak daha yüksek olduğu gözlendi (sırasıyla p≤ 0,001, <0,03, <0,001, 0,008).
*

**Sonuç:** *Ürik asit, total kolesterol ve GGT, OSAS'ta aralıklı hipoksiye bağlı olarak diğer sistemlerin de etkilenmesiyle artabilen biyobe- lirteçlerdir. Tedavi uyumuna bağlı olarak erken dönemde bu biyobelirteçlerde azalma gözlenebildiğinden, tedavi uyumu ve tedavi etkinliğinin takibinde bu biyobelirteçler pratik olarak kullanılabilir.
*

**Anahtar kelimeler:** *OSAS; pozitif hava yolu basıncı tedavisi; biyomarker
*

## INTRODUCTION

Obstructive sleep apnea syndrome (OSAS) is a clinical condition characterized by snoring, apnea, and hypopnea that develop due to recurrent upper respiratory tract obstruction during sleep. OSAS is a prevalent disease affecting approximately 13% of men and 6% of women, with an increasing prevalence in recent years (1).

The primary mechanism responsible for OSAS-related pathologies is increased oxidative stress due to the desaturation and reoxygenation cycle caused by apnea and hypopnea episodes, leading to endothelial damage and inflammation (2,3). The developing endothelial damage and inflammation can potentially affect almost all body systems, with particular impact on the cardiovascular system (4). One of the important consequences of oxidative stress in OSAS is that it leads to pyruvate accumulation by activating the glycolysis pathway. The accumulation of pyruvate and the release of purine intermediates leads to the overproduction of uric acid, the final breakdown product of purine metabolism. For this reason, tissue hypoxia may cause hyperuricemia in OSAS (5,6). In addition, studies have shown that OSAS may negatively affect renal function parameters other than uric acid and cause chronic renal failure due to effects such as hypoxia-induced increase in oxidative stress, activation of the renin-angiotensin-aldosterone system, or sympathetic activation (7).

OSAS can also negatively affect liver function. Recent studies have shown a relationship between OSAS, non-alcoholic fatty liver disease, and high liver enzymes (8). Studies argue that nocturnal hypoxic attacks, common in OSAS, are the primary mechanism that causes liver damage (9). Cases of ischemic hepatitis due to OSAS have also been reported, especially in obese patients with severe hypoxia (10,11). In addition to liver damage, OSAS may negatively affect lipid metabolism, causing dyslipidemia (12). Although the mechanism of dyslipidemia due to OSAS has not been fully explained, studies argue that hypoxia attacks, in particular, cause dyslipidemia by increasing oxidative stress (12).

Positive airway pressure therapy (PAP) is considered the gold standard treatment for OSAS as it can negatively affect nearly all body systems. PAP therapy alleviates symptoms and reduces complications by lessening apnea and hypopnea episodes through the delivery of airflow at a specified pressure (13). This treatment operates on the principle of delivering air pressure through a mask worn over the nose or mouth to prevent obstructions in the upper airway during sleep. The effectiveness of PAP treatment is closely tied to the patient’s adherence to the therapy. Treatment compliance involves using the device regularly and correctly according to the prescribed treatment plan. Adherence to PAP therapy is crucial for reducing symptoms, improving overall health, and

preventing long-term complications such as diabetes, hypertension, and coronary artery disease. Therefore, maintaining treatment adherence is essential for enhancing the quality of life and safeguarding patients’ health with OSAS (13).

In our study, it was aimed to examine the effects of treatment compliance on kidney and liver functions, the apnea-hypopnea index (AHI), and the lipid profile in patients diagnosed with OSAS via polysomnography (PSG) who had commenced PAP treatment.

## MATERIALS and METHODS

**Study Design**

Our study included 342 patients who applied to our hospital outpatient clinic between September 2022 and September 2023 with at least one of the complaints of witnessed apnea, snoring, and excessive daytime sleepiness, and subsequently admitted to our hospital’s sleep laboratory and underwent PSG. Our study was designed and conducted following the ethical principles of the Declaration of Helsinki. Written informed consent was obtained from all participants. The local ethics committee approved our study.

## Study Population

Among the 342 patients admitted to our hospital’s sleep laboratory, 64 patients with AHI< 5 and 72 who did not need to start PAP treatment despite having AHI> 5, were excluded from the study. In addition,

46 patients with known heart, lung, and kidney diseases, and 38 patients who had previously received PAP treatment, who used oral devices, and had surgery due to OSAS were excluded from the study. Furthermore, 44 patients with drug use and alcohol use, affecting BUN, creatinine, uric acid, cholesterol parameters, and liver enzymes in the month before PSG were also excluded from the study. During the control examination, patients whose body mass index (BMI) increased by more than 5% compared to the baseline, 12 patients who underwent surgery during the study period, and those and those taking medication that could affect kidney and liver functions due to newly diagnosed chronic disease were excluded from the study (Figure 1).

Considering these exclusion criteria, 66 patients were ultimately included in the study. Detailed anamnesis were taken from the patients in our study, along with a physical examination and anthropometric measurements were taken. The age and BMI of the

**Figure 1.** Consort diagram.

patients were recorded. Additionally, chest radiography and electrocardiogram were taken, and respiratory function tests were performed on the patients who did not have known pathologies to exclude unknown pathologies. Before PSG, blood was taken from the patients after at least eight hours of fasting, and serum uric acid, creatinine, BUN, HgbA1c, triglyceride, total cholesterol, LDL cholesterol, HDL cholesterol, ALT, AST, LDH, GGT, and ALP levels were recorded. The patients included in the study were categorized into three groups according to their AHI values after PSG. Patients with an AHI of 5-15 were grouped as mild, with AHI= 15-30 as moderate, and with AHI≥ 30 as having severe OSAS.

## Polysomnography

Complete polysomnography was monitored using the Compumedics E-series Sleep System (Compumedics Sleep, Melbourne, Vic., Australia). Electroencephalography (EEG), electrooculography, electromyography, and electrocardiography were performed simultaneously. Surface electrodes were used to record EEG channels, right and left electrooculography, and submental electromyography. Ventilatory flow was measured with airflow, either at the nose or the nose and the mouth. Respiratory movements of the chest and abdomen, as well as the body position, were monitored by inductive plethysmography bands. Arterial oxygen saturation was measured transcutaneously with a finger oximeter. Apnea was defined as continuous cessation of airflow for ≥10 s, and hypopnea was defined as at least 30% reduction of airflow for ≥10 s with an oxygen desaturation of ≥3% or an EEG arousal from sleep. Apneas were classified as obstructive, central, or mixed according to the standard criteria of the American Academy of Sleep Medicine (14).

## Follow-ups

The patients included in the study were called for examination six months after starting PAP treatment. PAP compliance was estimated by dividing the total hours recorded on the device timer by the number of nights between the start of treatment and follow-up examination. Compliance with PAP therapy was characterized by at least four hours per night and the use of the device more than 70% of nights (15). Patients with PAP compliance were called group 1, and patients without PAP compliance were called group 2.

## Laboratory Measurements

Aspartate aminotransferase (AST, U/L), alanine ami- notransferase (ALT, U/L), alkaline phosphatase (ALP U/L), gamma glutamine transpeptidase (GGT, U/L), lactate dehydrogenase (LDH, U/L), urea (mg/dL), total cholesterol (mg/dL), high-density lipoprotein-choles- terol (HDL-C, mg/dL), low-density lipoprotein-cho- lesterol (LDL-C, mg/dL) and triglycerides (mg/dL) serum levels were analyzed via spectrophotometric method in Beckman Coulter AU 5800 (Beckman Coulter, CA, USA) auto analyzer. Uric acid (mg/dL) was measured on a Beckman Coulter AU-5800 instrument (Beckman Coulter, CA, USA) by the uricase-peroxidase method. The Jaffe method assessed creatinine (mg/dL) on a Beckman Coulter AU 5800 instrument (Beckman Coulter, CA, USA). Blood urea nitrogen (BUN, mg/dL) levels were calcu- lated from urea values (BUN= urea/2.14). High- performance liquid chromatography (HPLC) method was used for HbA1c (%) (Lifotronic H9, Lifotrophic Technology, Shenzhen, China).

## Statistics Analysis

The G Power program was utilized to determine the number of patients required before initiating the study. Based on our hypothesis, the study “Serum uric acid and arterial lactate levels in patients with obstructive sleep apnea syndrome: the effect of CPAP treatment” was referenced to calculate our effect size. Consequently, our effect size was established at 1.81, with α= 0.05 and 1-β= 0.95, leading to a calculated minimum sample size of 16. Data analyses were conducted using the IBM SPSS Statistics version

20.0 software (IBM Corp., Armonk, NY). The Kolmogorov-Smirnov test was employed to ascertain the normal distribution of the data under study. The Mann-Whitney U test was applied to compare baseline and six-month laboratory parameters of the patients. Changes in PSG data over the six months of treatment were compared using a paired-sample t-test. An independent-sample t-test was used for comparing changes in laboratory and PSG data at the six-month follow-up. Spearman’s correlation analysis facilitated the evaluation of correlations between laboratory parameters. P values <0.05 were considered statistically significant.

## RESULTS

Mean age of the patients in our study was 54.9 ±

14.1 years. Mean age of the patients who used PAP

treatment by the criteria we determined were 55.5 ± 15.6, while the mean age of the patients who did not use it was 54.1 ± 12.3. No significant difference was observed in the statistical analysis between the groups (p= 0.67). Of the patients included in our study, 35 (53%) were males. In the statistical analysis made according to sex, it was observed that there was no significant difference (p= 0.72).

The laboratory data and polysomnographic analysis of the patients that were followed up with a diagnosis of OSAS at baseline and six months later are shown in Table 1. Accordingly, it was observed that uric acid, BUN, triglyceride, total cholesterol, ALT, GGT, ALP, and AHI levels decreased during follow-up (p= 0.001, 0.006, <0.001, 0.006, 0.01, <0.001, <0.001,

<0.001, respectively). It was observed that HDL cholesterol levels increased (p≤ 0.001). The changes in laboratory and polysomnographic data between group 1 and group 2 patients over six months are shown in Table 2. Accordingly, it was observed that the change in uric acid, AHI, total cholesterol, and GGT levels in group 1 patients was statistically higher than in group 2 patients (p≤ 0.001, <0.03,

<0.001, 0.008, respectively).

The comparison of the oxygen desaturation index (ODI), mean oxygen saturation, and minimum oxygen saturation of OSAS patients before and after

treatment is shown in Table 3. Accordingly, mean and minimum oxygen saturation increased, and ODi levels decreased in both groups (p≤ 0.001 for all). However, no significant difference was observed between the groups when the six-month parameter changes were compared (p≥ 0.05 for all). In the evaluation of the comorbidities of the patients included in our study, 14 patients (21.2%) had diabetes mellitus (DM) and 16 patients (24.2%) had hypertension (HT). In evaluating the initial laboratory parameters of the patients with DM, it was observed that uric acid, HgbA1c, and LDH levels were statistically significantly higher (p= 0.02, <0.001, 0.02, respectively). In patients with HT, triglyceride, total cholesterol, and LDL levels were statistically significantly higher (p≤ 0.001, 0.01, 0.02, respectively).

Correlation analysis of the changes in laboratory data and polysomnographic data after six months of treatment is given in Table 4. Accordingly, no significant correlation was observed between AHI, Δ ODI, Δ mean oxygen saturation, and Δ minimum oxygen saturation and laboratory data. However, a positive correlation was observed between Δ total cholesterol and Δ uric acid and Δ triglyceride (r= 0.366, p= 0.01, r= 0.421, p= 0.01, respectively)

(Figure 2).

**Table:** 

**Table 1.** Comparison of laboratory and polysomnographic data at baseline and sixth month of treatment			
	**Before treatment**	**After six months follow-up**	**p**
AHI	42.7 ± 30	9.9 ± 10.9	<0.001
Uric acid	6 ± 1.7	5.5 ± 1.5	0.001
Creatinine	0.8 ± 0.2	0.8 ± 0.2	0.41
BUN	16.3 ± 7.1	14.9 ± 5.3	0.006
HgbA1c	6.2 ± 1.1	6.2 ± 1.2	0.6
TG	242.1 ± 136.1	194.9 ± 119.4	<0.001
Total cholesterol	202.8 ± 42.9	187.5 ± 44.7	0.006
LDL	134.6 ± 33.7	130.2 ± 32.7	0.43
HDL	41.1 ± 13.7	45.2 ± 11.9	<0.001
ALT	29.2 ± 17.9	24.8 ± 14.1	0.014
AST	24.5 ± 11.2	21.8 ± 6.8	0.13
LDH	218.8 ± 65.1	223.4 ± 84.9	0.91
GGT	39.9 ± 23.7	34.4 ± 23.6	<0.001
ALP	92.1 ± 34.3	80.9 ± 37.4	<0.001
AHI: Apnea hypopnea index, TG: Triglyceride, AST: Aspartate aminotransferase, ALT: Alanine aminotransferase, ALP: Alkaline phosphatase, GGT: Gamma glutamine transpeptidase, LDH: Lactate dehydrogenase, HDL: High-density lipoprotein, LDL: Low-density lipoprotein.			

**Table:** 

**Table 2.** Changes in laboratory and polysomnographic data between group 1 and group 2 patients in over six months					
	**Groups**	**n**	**Mean**	**Std. Deviation**	**p**
	Group 2	30	-0.25	1.19	
Δ Uric acid					<0.001
	Group 1	36	1,21	1.09	
	Group 2	30	24.57	21.40	
Δ AHI					0.03
	Group 1	36	39.60	30.10	
	Group 2	30	33.80	126.96	
ΔTG					0.4
	Group 1	36	58.33	105.27	
	Group 2	30	-7.03	40.48	
Δ Total cholesterol					<0.001
	Group 1	36	33.86	39.56	
	Group 2	30	-2.93	7.01	
Δ HDL					0.36
	Group 1	36	-5.06	10.88	
	Group 2	30	1.30	9.90	
Δ ALT					0.14
	Group 1	36	7,03	18.90	
	Group 2	30	1.50	6.46	
Δ GGT					0.008
	Group 1	36	8.92	25.22	
Group 1: PAP-compliant patients, Group 2: PAP incompliant patients, AHI: Apnea hypopnea index, TG: Triglyceride, AST: Aspartate aminotrans- ferase, ALT: Alanine aminotransferase, ALP: Alkaline phosphatase, GGT: Gamma glutamine transpeptidase, LDH: Lactate dehydrogenase, HDL: High-density lipoprotein, LDL: Low-density lipoprotein.					

**Table:** 

**Table 3.** Comparison of polysomnographic data of the groups before and after treatment and changes with treatment						
		**Group 1**		**Group 2**		
	**Before Treatment**	**After six months follow-up**	p*	** Before After six months Treatment follow-up **	p*	
ODI	6.1 ± 2.9	5.1 ± 2.4	<0.001	6.4 ± 2.7 5.1 ± 2.4	<0.001	
Mean O2	83.8 ± 7.4	90.1 ± 3.1	<0.001	84.2 ± 4.9 89.6 ± 2.8	<0.001	
Min. O2	71.6 ± 11.4	82.2 ± 5.5	<0.001	69.4 ± 9.2 80.7 ± 5.4	<0.001	p**
Δ ODI		1.5 ± 2.2	N/A	1.2 ± 1.4	N/A	0.61
Δ Mean O2		6.3 ± 5.2	N/A	5.4 ± 3.6	N/A	0.98
Δ Min. O2		10.5 ± 7.6	N/A	11.3 ± 6.5	N/A	0.48
ODI: Oxygen desaturation index, Min: Minimum, p*: Comparison of polysomnographic data at six-month follow-up within the groups. p**: Comparison of the changes in polysomnographic data between the groups after six months of follow-up.						

## DISCUSSION

In our study, at the end of the sixth month, patients who began PAP treatment were found to have decreased levels of uric acid, BUN, triglyceride, total cholesterol, ALT, GGT, ALP, and AHI, while their HDL levels increased. It was observed that the changes in uric acid, AHI, total cholesterol, and GGT levels were significantly greater in patients who adhered to PAP treatment compared to those who were non- compliant. Correlation analysis of the laboratory data showed a positive correlation between the decrease in total cholesterol level and the decrease in uric acid and triglyceride levels after six months of follow-up.

OSAS is a syndrome that primarily affects many organs and systems due to intermittent hypoxia and increased intrathoracic pressure, caused mainly by the anatomically narrow and collapsed oropharynx. Without appropriate treatment, oxidative stress may lead to severe comorbidities, including endothelial damage, inflammation, and increased sympathetic system activity (16).

In OSAS, biochemical parameters can vary, increase or decrease depending on the affected organs and systems. An activation of the glycolysis pathway due to increased oxidative stress might lead to an accumulation and subsequent increase in uric acid.

**Table:** 

**Table 4.** Correlation of the changes observed in laboratory parameters within six months											
		**Δ Uric acid**	**Δ AHI**	**Δ TG**	**Δ Total Cholesterol**	**Δ HDL**	**Δ ALT**	**Δ GGT**	**Δ ODI**	**Δ Mean O2**	**Δ Min. O2**
Δ AHI	R p	0.10 0.43	1								1 .
Δ TG	R p	0.21 0.09	0.08 0.52	1							
Δ Total cholesterol	R p	0.366** 0.00	0.22 0.08	0.421** 0.00	1						
Δ HDL	R p	-0.11 0.39	-0.18 0.15	0,09 0.47	-0.08 0.52	1					
Δ ALT	R p	-0.11 0.40	-0.01 0.91	-0.04 0.72	0.14 0.26	0.11 0.39	1				
Δ GGT	R p	0.02 0.90	0.02 0.86	-0.07 0.60	-0.06 0.65	-0.01 0.96	0.10 0.45	1			
Δ ODI	R p	-0.20 0.10	0.13 0.28	0.07 0.54	-0.10 0.42	-0.07 0.59	-0.20 0.10	0.15 0.22	1 .		
Δ Mean O2	R p	-0.07 0.58	0.14 0.26	-0.06 0.63	0.09 .455	-0.17 0.16	-0.02 0.86	0.09 0.45	0.27* 0.03	1 .	
Δ Min. O2	R p	-0.18 0.15	0.30* 0.01	0.01 0.93	0.03 0.80	-0.17 0.17	-0.06 0.62	0.06 0.59	0.46** 0.00	0.69** 0.00	
AHI: Apnea hypopnea index, TG: Triglyceride, ALT: Alanine aminotransferase, GGT: Gamma glutamine transpeptidase, HDL: High-density lipopro- tein, ODI: Oxygen desaturation index, Min: Minimum, *Correlation is significant at the 0.05 level (2-tailed),**Correlation is significant at the 0.01 level (2-tailed).

**Figure 2.** Correlation analysis between Δ total cholesterol and Δ uric acid, Δ triglycerides.

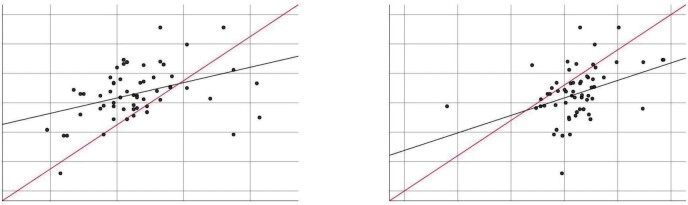
Impairment of kidney functions, possibly due to the activation of the renin-angiotensin-aldosterone system, can result in elevated BUN creatinine levels and reduced glomerular filtration rate (GFR) (5,7). The development of hypoxia-related non-alcoholic fatty liver disease might cause an increase in liver enzymes (8). Additionally, OSAS may disrupt the lipid profile in the blood, leading to dyslipidemia (12). Monitoring

these changes in biochemical parameters is crucial for determining the severity of the disease and assessing the response to treatment.

One study assessed uric acid levels before and after PAP treatment in patients diagnosed with OSAS. This study revealed a significant decrease in uric acid levels in patients who were compliant with PAP

treatment compared to non-compliant individuals (17). These findings are consistent with our research, suggesting that high uric acid levels result from increased oxidative stress due to nocturnal hypoxia. Considering uric acid as an established biomarker in cardiovascular diseases, adherence to PAP treatment could potentially reduce cardiovascular comorbidities associated with OSAS (18). Another study has focused on the relationship between OSAS severity and uric acid levels, finding a direct correlation with AHI (3). Further, research examining uric acid levels in OSAS patients has identified a positive correlation with the extent of hypoxia (19). These studies collectively associate increased uric acid with nocturnal hypoxia.

One study investigating the effect of OSAS on liver enzymes has found that ALT and GGT are higher in patients diagnosed with OSAS compared to the average population. In particular, it has been observed that GGT increases in correlation with the OSAS degree. This study has concluded that GGT is more affected by OSAS than other liver enzymes. Another study has investigated the relationship of ALT and AST with OSAS severity and PSG parameters. In this study, elevation of aminotransferases has been significantly associated with the severity of OSAS, but no significant relationship has been found between them and PSG parameters. Elevated liver enzymes in these patients are primarily attributed to mitochondrial anaerobic respiration and catecholamine-mediated metabolic changes triggered by hypoxia, as indicated in the studies (20- 22). In another study, the effect of PAP compliance on ALT and AST levels has been investigated in patients diagnosed with OSAS following a four-week follow-up. The study has concluded that although there was a decrease in aminotransferase levels in patients who started PAP treatment, PAP compliance was not associated with the reduction in aminotransferase levels (23). The short patient follow- up period was the most important limitation of this study. In our study, the decrease in liver enzyme levels such as ALT, AST, and GGT in patients undergoing PAP therapy may indicate that PAP therapy could positively affect liver health. This finding suggests that the adverse effects of OSAS on the liver, characterized by increased systemic inflammation and oxidative stress, may be reduced by PAP therapy. In addition, the decrease in GGT level was more pronounced in patients undergoing PAP therapy in our study compared to those in the

literature, which may be attributed to the fact that GGT is more susceptible to oxidative stress than other liver enzymes (20). Studies have shown that GGT is more sensitive to oxidative stress than other liver enzymes. GGT is expected to be the first enzyme to recover due to the decrease in oxidative stress, especially in patients who adhere to PAP treatment. Considering that systemic effects caused by OSAS may lead to more rapid and measurable changes in GGT than in other liver enzymes, GGT may be a more sensitive marker than other liver enzymes in evaluating metabolic and functional responses to PAP therapy.

OSAS is considered an independent risk factor for dyslipidemia. Studies have shown that the inflammation and oxidative stress that develop due to intermittent hypoxia can disrupt lipid metabolism (24,25). Although the results of the studies vary, triglyceride, total cholesterol, and LDL levels were found to be high in a significant portion of OSAS patients, while HDL levels were low (26,27). The results of studies examining the effect of PAP treatment on the lipid profile vary. One study has concluded that PAP treatments significantly reduce total cholesterol levels, especially in young and obese patients, but has no effect on triglyceride, LDL, and HDL levels. In another study, a decrease has been noted in triglyceride, total cholesterol, and LDL levels in patients adhering to PAP treatment, while HDL levels have shown an increase (28,29). In our study, we attribute the decrease in triglyceride and total cholesterol levels, and the increase in HDL levels in patients receiving PAP therapy, to the fact that PAP therapy prevents airway obstruction and hypoxia that develop during sleep. Additionally, the increased metabolic rate due to decreased hypoxia may also favorably affect lipid metabolism. The decrease in triglyceride and total cholesterol levels may be associated with the regulating effect of PAP treatment on carbohydrate and fatty acid metabolism. Accordingly, the body’s enhanced energy use can help to reduce the storage of lipids. These findings suggest that PAP therapy not only improves respiratory function but also has far-reaching favorable effects on the metabolic profile and can potentially reduce cardiovascular risk in patients with OSAS. The significantly greater decrease in total cholesterol levels in patients who adhered to PAP treatment, compared to the other group, may be attributed to the fact that total cholesterol is more comprehensively

affected by treatment as a parameter that reflects both anabolic and catabolic effects in general. Furthermore, it has also been shown in the literature that total cholesterol responds earlier to oxidative stress and inflammation than other lipids (30).

Although many surgical and medical methods can be used to treat OSAS, the gold standard in treatment is PAP therapy. The primary purpose of PAP therapy is to ensure that the airways, which collapse during sleep, remain open with pressurized airflow (31). Thus, it aims to prevent complications by reducing nocturnal hypoxia, which plays a fundamental role in the pathogenesis of OSAS (31). In our study, examining the effect of compliance with PAP therapy on biochemical parameters, it was observed that uric acid, BUN, triglyceride, total cholesterol, ALT, and GGT levels decreased significantly at the end of the sixth month in all patients who started PAP therapy. Moreover, in contrast, uric acid and total cholesterol levels in patients who adhered to the therapy decreased compared to the other group, with a significantly greater decrease in GGT levels. The decrease in uric acid and BUN levels may be due to the reduction of nocturnal hypoxia and the resulting decline in oxidative stress. After the reduction in uric acid level, which is used as a cardiac biomarker in particular, the benefits of PAP therapy on cardiac health become more evident. In our study, the decrease in ALT and GGT levels in patients receiving PAP therapy may be due to the reduced mitochondrial anaerobic respiration caused by the decline in hypoxemia. ALT and GGT are enzymes that increase in non-alcoholic fatty liver disease. The decrease in ALT and GGT levels may indicate that PAP therapy may reduce the incidence of non-alcoholic fatty liver disease. Since GGT levels decrease more in patients who are compliant with treatment, GGT may be a more sensitive biomarker than other liver enzymes in monitoring the effectiveness of PAP therapy. Additionally, studies have shown that an elevation in GGT is an independent risk factor for cardiac diseases and hypertension in OSAS (32,33). The significant decrease in GGT levels in patients compliant with treatment can be interpreted as an indication that compliance with treatment may reduce the risk of cardiac complications and hypertension. One of the significant findings of our study is the effect of PAP therapy on the lipid profile. The decrease in total cholesterol and triglyceride

levels, along with the increase in HDL levels in patients receiving PAP therapy, may be attributed to reduced inflammation and oxidative stress secondary to hypoxemia. The positive effects of PAP therapy on the lipid profile suggest that the treatment reduces the risk of atherosclerosis. Furthermore, the significant decrease in total cholesterol levels in patients using PAP therapy compliantly may be attributed to the fact that total cholesterol responds more promptly to the reduction of oxidative stress and inflammation than other lipids (30). In evaluating compliance with treatment and the therapy’s effect on dyslipidemia in OSAS patients, total cholesterol may be a more sensitive biomarker than other lipids, particularly at the onset of treatment.

The most significant limitation of our study is the inclusion of some patients with comorbidities such as DM and HT. Although patients on medications that could potentially affect our study’s results were excluded, these comorbidities themselves might still influence the findings. Additionally, our study’s duration was limited to six months. Extending the follow-up period would allow a more comprehensive evaluation of the biochemical changes related to the response to treatment.

As a result, our study found that the change in uric acid, total cholesterol, and GGT levels was more significant in patients who used PAP treatment compliantly compared to the other group. Considering these biomarkers are significantly associated with cardiac pathologies, treatment compliance may reduce cardiac comorbidities in OSAS. Given that elevations in uric acid and GGT can be attributable to other systems, especially the renal and hepatobiliary systems, compliance with PAP treatment may also mitigate the negative impact of OSAS on these systems. Additionally, these biomarkers can be utilized to monitor treatment compliance. More comprehensive studies, with longer follow-up periods and additional parameters, are needed to further examine the effect of compliance with PAP treatment on biochemical parameters.

**Ethical Committee Approval:** This study was approved by the Atatürk University Faculty of Medicine Clinical Research Ethics Committee (Decision no: B.30.2.ATA.0.01.00/847, Date: 26.10.2023).

## CONFLICT of INTEREST

The authors declare that they have no conflict of interest.

## AUTHORSHIP CONTRIBUTIONS

Concept/Design: AA, BK Analysis/Interpretation: BK, EYU Data acqusition: HBÖ, ÖA, EL Writing: AA

Clinical Revision: AA, ÖA, BK Final Approval: LS, EYU, ÖA

## REFERENCES

1. *
  Heinzer R, Vat S, Marques-Vidal P, Marti-Soler H, Andries D, Tobback N, et al. Prevalence of sleep-disordered breathing in the general population: The HypnoLaus study. Lancet Respir Med 2015; 3(4): 310-8. https://doi. org/10.1016/S2213-2600(15)00043-0*
2. *
  Peppard PE, Young T, Barnet JH, Palta M, Hagen EW, Hla KM. Increased prevalence of sleep-disordered breathing in adults. Am J Epidemiol 2013; 177(9): 1006-14. https:// doi.org/10.1093/aje/kws342*
3. *
  Hirotsu C, Tufik S, Guindalini C, Mazzotti DR, Bittencourt LR, Andersen ML. Association between uric acid levels and obstructive sleep apnea syndrome in a large epidemiolog- ical sample. PloS One 2013. 8(6): e66891. https://doi. org/10.1371/journal.pone.0066891*
4. *
  Vijayan VK. Morbidities associated with obstructive sleep apnea. Expert Rev Respir Med 2012; 6(5): 557-66. https://doi.org/10.1586/ers.12.44*
5. *
  Bender DA, Mayes PA. Bender, glycolysis and the oxida- tion of pyruvate. a LANGE medical book, 2003: 136.
  *
6. *
  Kreisberg RA. Lactate homeostasis and lactic acidosis. Ann Intern Med 1980; 92(2 Pt 1): 227-37. https://doi. org/10.7326/0003-4819-92-2-227*
7. *
  Kanbay A, Buyukoglan H, Ozdogan N, Kaya E, Oymak FS, Gulmez I, et al. Obstructive sleep apnea syndrome is relat- ed to the progression of chronic kidney disease. Int Urol Nephrol 2012; 44(2): 535-9. https://doi.org/10.1007/ s11255-011-9927-8*
8. *
  Tanné F, Gagnadoux F, Chazouillères O, Fleury B, Wendum D, Lasnier E, et al. Chronic liver injury during obstructive sleep apnea. Hepatology 2005; 41(6): 1290-6. https:// doi.org/10.1002/hep.20725*
9. *
  Mishra P, Nugent C, Afendy A, Bai C, Bhatia P, Afendy M, et al. Apnoeic-hypopnoeic episodes during obstructive sleep apnoea are associated with histological nonalcohol- ic steatohepatitis. Liver Int 2008; 28(8): 1080-6. https:// doi.org/10.1111/j.1478-3231.2008.01822.x*
10. *
  Henrion J, Colin L, Schapira M, Heller FR. Hypoxic hepa- titis caused by severe hypoxemia from obstructive sleep apnea. J Clin Gastroenterol 1997; 24(4): 245-9. https:// doi.org/10.1097/00004836-199706000-00013*
11. *
  Trakada G, Gogos C, Tsiamita M, Siagris D, Goumas P, Spiropoulos K. A case of ischemic hepatitis. Sleep Breath 2004; 8(3): 155- 9. https://doi. org/10.1055/s-2004-834486*
12. *
  Adedayo AM, Olafiranye O, Smith D, Hill A, Zizi F, Brown C, et al. Obstructive sleep apnea and dyslipidemia: Evidence and underlying mechanism. Sleep Breath 2014; 18(1): 13-8. https://doi.org/10.1007/s11325-012-0760- 9*
13. *
  Pavwoski P, Shelgikar AV. Treatment options for obstruc- tive sleep apnea. Neurol Clin Pract 2017; 7(1): 77-85. https://doi.org/10.1212/CPJ.0000000000000320*
14. *
  Ruehland WR, Rochford PD, O’Donoghue FJ, Pierce RJ, Singh P, Thornton AT. The new AASM criteria for scoring hypopneas: Impact on the apnea hypopnea index. Sleep 2009; 32(2): 150-7. https://doi.org/10.1093/ sleep/32.2.150*
15. *
  Kapur VK, Auckley DH, Chowdhuri S, Kuhlmann DC, Mehra R, Ramar K, et al. Clinical practice guideline for diagnostic testing for adult obstructive sleep apnea: An American Academy of sleep medicine clinical practice guideline. J Clin Sleep Med 2017; 13(3): 479-504. https://doi.org/10.5664/jcsm.6506*
16. *
  Dempsey JA, Veasey SC, Morgan BJ, O’Donnell CP. Pathophysiology of sleep apnea. Physiol Rev 2010; 90(1): 47-112. https://doi.org/10.1152/physrev.00043.2008*
17. *
  Bartziokas K, Papaioannou AI, Haniotou A, Nena E, Kostikas K, Steiropoulos P. Serum uric acid and arterial lactate levels in patients with obstructive sleep apnea syndrome: The effect of CPAP treatment. Postgrad Med 2021; 133(5): 518-24. https://doi.org/10.1080/0032548 1.2020.1866891*
18. *
  Kanbay A, Inonu H, Solak Y, Erden A, Uslu E, Yuksel SA, et al. Uric acid as a potential mediator of cardiovascular morbidity in obstructive sleep apnea syndrome. Eur J Intern Med 2014; 25(5): 471-6. https://doi.org/10.1016/j. ejim.2014.04.005*
19. *
  Hira HS, Shukla A, Kaur A, Kapoor S. Serum uric acid and lactate levels among patients with obstructive sleep apnea syndrome: Which is a better marker of hypoxemia? Ann Saudi Med 2012; 32(1): 37-42. https://doi. org/10.5144/0256-4947.2012.37*
20. *
  Li J, Zhang YL, Chen R, Wang Y, Xiong KP, Huang JY, et al. Elevated serum liver enzymes in patients with obstructive sleep apnea-hypopnea syndrome. Chin Med J (Engl) 2015; 128(22): 2983-7. https://doi.org/10.4103/0366- 6999.168943*
21. *
  Atan D, Köseoğlu S, Özcan KM, İkincioğulları A, Topak AB, Özcan İ, et al. Evaluation of liver functions based on serum aminotransferase enzyme levels in patients with obstructive sleep apnea syndrome. Indian J Otolaryngol Head Neck Surg 2019; 71(Suppl 3): 1679-82. https://doi. org/10.1007/s12070-015-0914-8*
22. *
  Sookoian S, Rosselli MS, Gemma C, Burgueño AL, Fernández Gianotti T, Castaño GO, et al. Epigenetic regu- lation of insulin resistance in nonalcoholic fatty liver dis- ease: Impact of liver methylation of the peroxisome prolif- erator-activated receptor* γ *coactivator 1 *α *promoter. Hepatology 2010; 52(6): 1992-2000. https://doi. org/10.1002/hep.23927*
23. *
  Kohler M, Pepperell JC, Davies RJ, Stradling JR. Continuous positive airway pressure and liver enzymes in obstructive sleep apnoea: Data from a randomized controlled trial. Respiration 2009; 78(2): 141-6. https://doi. org/10.1159/000170785*
24. *
  Kono M, Tatsumi K, Saibara T, Nakamura A, Tanabe N, Takiguchi Y, et al. Obstructive sleep apnea syndrome is associated with some components of metabolic syn- drome. Chest 2007; 131(5): 1387-92. https://doi. org/10.1378/chest.06-1807*
25. *
  Börgel J, Sanner BM, Bittlinsky A, Keskin F, Bartels NK, Buechner N, et al. Obstructive sleep apnoea and its ther- apy influence high-density lipoprotein cholesterol serum levels. Eur Respir J 2006; 27(1): 121-7. https://doi. org/10.1183/09031936.06.00131304*
26. *
  Li J, Savransky V, Nanayakkara A, Smith PL, O’Donnell CP, Polotsky VY. Hyperlipidemia and lipid peroxidation are dependent on the severity of chronic intermittent hypoxia. J Appl Physiol (1985) 2007; 102(2): 557-63. https://doi. org/10.1152/japplphysiol.01081.2006*
27. *
  Gündüz C, Basoglu OK, Hedner J, Zou D, Bonsignore MR, Hein H, et al. Obstructive sleep apnoea independently predicts lipid levels: Data from the European Sleep Apnea Database. Respirology 2018; 23(12): 1180-9. https://doi. org/10.1111/resp.13372*
28. *
  Dorkova Z, Petrasova D, Molcanyiova A, Popovnakova M, Tkacova R. Effects of continuous positive airway pressure on cardiovascular risk profile in patients with severe obstructive sleep apnea and metabolic syndrome. Chest 2008; 134(4): 686-92. https://doi.org/10.1378/chest.08- 0556*
29. *
  Hofer P, Boeszoermenyi A, Jaeger D, Feiler U, Arthanari H, Mayer N, et al. Fatty acid-binding proteins interact with comparative gene identification-58 linking lipolysis with lipid ligand shuttling. J Biol Chem 2015; 290(30): 18438- 53. https://doi.org/10.1074/jbc.M114.628958*
30. *
  Robinson GV, Pepperell JC, Segal HC, Davies RJ, Stradling JR. Circulating cardiovascular risk factors in obstructive sleep apnoea: Data from randomised controlled trials. Thorax 2004; 59(9): 777-82. https://doi.org/10.1136/ thx.2003.018739*
31. *
  Patil SP, Ayappa IA, Caples SM, Kimoff RJ, Patel SR, Harrod CG, et al. Treatment of adult obstructive sleep apnea with positive airway pressure: An American Academy of sleep medicine clinical practice guideline. J Clin Sleep Med 2019; 15(2): 335-43. https://doi.org/10.5664/jcsm.7640*
32. *
  Kanbay A, Kaya E, Buyukoglan H, Ozdogan N, Kaya MG, Oymak FS, et al. Serum gamma-glutamyl transferase activ- ity is an independent predictor for cardiovascular disease in obstructive sleep apnea syndrome. Respir Med 2011; 105(4): 637-42. https://doi.org/10.1016/j. rmed.2010.12.003*
33. *
  Bozkus F, Dikmen N, Demir LS. Gamma-glutamyl transfer- ase activity as a predictive marker for severity of obstruc- tive sleep apnea syndrome and concomitant hyperten- sion. Clin Respir J 2018; 12(5): 1964-73. https://doi. org/10.1111/crj.12765*

